# The Impact of Dental Pain on the Oral Health-Related Quality of Life (OHRQoL) of Preschool Children in Austria

**DOI:** 10.3390/jcm12185906

**Published:** 2023-09-11

**Authors:** Sophie Lembacher, Viktoria Hofer, Katrin Bekes

**Affiliations:** Department of Paediatric Dentistry, University Clinic of Dentistry, Medical University Vienna, Sensengasse 2a, 1090 Vienna, Austria; sophie.lembacher@meduniwien.ac.at (S.L.); viktoria.hofer@meduniwien.ac.at (V.H.)

**Keywords:** dental pain, oral health, quality of life, ECOHIS

## Abstract

Dental pain in children is a global public health burden with psychosocial and economic implications, challenging families and pediatric dentists in daily clinical practice. Previous studies have addressed the exclusive impact of either caries, dental trauma, malocclusion, or socioeconomic status on OHRQoL. Even though such examples can surely cause dental pain in children, so far only little research on the correlation of dental pain as a general symptom of different underlying causes and OHRQoL has been published. The aim of this study was to evaluate the impact of dental pain on the oral health-related quality of life (OHRQoL) of children between the ages of 0 and 6 years old and subsequently compare the results to a control group free of tooth ache. Children and their adult caregivers were recruited from the Emergency unit of the Department of Pediatric Dentistry at the University Clinic of Dentistry in Vienna. The caregivers completed the German version of the Early Childhood Oral Health Impact Scale (ECOHIS-G). Afterwards, the children were clinically examined. The cause for dental pain, dmf-t index, and plaque accumulation were collected. In total, 259 children with a mean age of 4.2 years (SD ± 1.5 years) were included in the study group. Their mean ECOHIS-G score was 9.0 (SD ± 7.4), while the control group only amounted to a score of 4.9 (SD ± 5.6). The difference between the two groups was statistically significant in both ECOHIS-G subsections, the child impact scale (CIS) and the family impact scale (FIS) as well as the ECOHIS-G sum score (*p* < 0.05). Dmf-t index and plaque accumulation significantly correlated with CIS and ECOHIS sum score (*p* ≤ 0.05). The reduction in quality of life was nearly twice as great in the children with dental pain as in the control children. The ECOHIS-G is a valid instrument for measuring the OHRQoL of children with dental pain between the ages of 0 to 6.

## 1. Introduction

Dental pain in children is a global public health burden with psychosocial and economic implications, challenging families and pediatric dentists in daily clinical practice. According to the International Association for the Study of Pain (ISAP) the updated definition of pain reads as follows: “Pain is unpleasant sensory and emotional experience associated with, or resembling that associated with, actual or potential tissue damage” [1]. Dental pain is described as pain originating from innervated dental tissues or tissues immediately adjacent to the teeth [2]. Estimations on prevalence of dental pain in children and adolescents range from 9.1% and 58% [3,4,5,6]. So far, no national data on the prevalence of tooth ache and population-based studies analyzing associated factors with dental pain in preschool children in Austria has been published.

The prevention and adequate management of dental pain requires knowledge on Its predictors. Numerous studies have examined social and demographic determinants and established a strong association between dental pain in children and the family’s socioeconomic background [7,8]. Acknowledging that various disease patterns can lead to tooth ache, the vast majority of cases are, however, caused by tooth decay and its complications. Dental caries is the most common human infective disease, affecting 30–90% of preschool children worldwide [9,10,11]. A severe and specific form of dental decay in the primary dentition of infants and preschool children is known as Early Childhood Caries (ECC). It is a particularly aggressive form of caries, beginning soon after dental eruption, progressing rapidly and often affecting immediate and long-term quality of life of the child and their family [12]. The multifactorial etiology is linked to breastfeeding or bottle nursing ad libitum, an increased intake of sugars, and poor oral hygiene [13]. Even though the global prevalence of ECC has decreased in recent years, it continues to be a serious public health problem [14]. The prevalence of ECC in developing countries and certain subpopulations within industrialized nations such as rural communities and the migrant communities may reach up to 90% [15]. Affected children frequently require treatment under general anesthesia, as their level of compliance and concentration span are often too little to proceed with ambulatory treatment. This has considerable costs and social implications [16]. Further, missed work days due to filial leaves and additional expenses for dental care often strain parents and caregivers [16,17]. This shows that the impact ECC has on children and their families is wide ranged, reaching from oral symptoms to behavioral changes and developmental problems of the child to socioeconomic challenges for families and communities. Consequently, the quality of life of children and their families can be severely compromised.

The concept of oral health-related quality of life (OHRQoL) has been established as an essential measure to examine the impact of oral conditions in children and adults on the individual’s psychosocial well-being [18]. By including the patient’s or parent’s self-perceived oral health and needs in terms of social or psychological impacts, OHRQoL allows for a comprehensive assessment of the patients’ oral health complementing traditional diagnostic criteria in clinical practice [19]. One of the most used instruments to assess OHRQoL in preschool children is the Early Childhood Oral Health Impact Scale (ECOHIS), a questionnaire specifically developed for application in epidemiological surveys [20]. While self-reports on one’s personal pain have been established as the gold standard in quantifying pain [21], limited communication skills and not yet fully developed cognitive capacity of infants and preschool children call for a different approach when analyzing the impact of dental pain on their mental health and social well-being. Therefore, OHRQoL of preschoolers is measured by proxy administration through letting their parents or caregivers answer the questionnaire for them. Otherwise, an underestimation of the impact of children’s oral health problems would be a valid risk in gaining a representative evaluation of the matter [18]. The ECOHIS is a scientifically validated instrument and has been translated into several different languages allowing the comparison of OHRQoL of children in various countries [22,23,24,25,26]. The German version of ECOHIS, ECOHIS-G, was developed and validated in 2019 at the University Clinic of Dentistry in Vienna [27]. Traditionally, ECOHIS is used to analyze children from 0 to 5 years old. However, the questionnaire has also been applied for 6-year-old children.

Previous studies have addressed the exclusive impact of either caries, dental trauma, malocclusion, or socioeconomic status on OHRQoL [12,28]. Even though such examples can surely cause dental pain in children, so far only little research on the correlation of dental pain as a general symptom of different underlying causes and OHRQoL has been published. Research assessing possible associated factors for the occurrence of dental pain and its impact on OHRQoL is scarce. As such knowledge is considered helpful to develop public health strategies targeting populations with higher risk of dental pain, the presented study aims to analyze the impact of dental pain on the OHRQoL of preschool children from 0 to 6 years old, assess associated factors, and compare their results with children of the same age range free of tooth ache.

## 2. Methods

The presented study is a population-based controlled study that assessed the impact of dental pain, caries experience, plaque, oral health, and overall well-being on the OHRQoL. Children and their caregivers were recruited from the Emergency unit of the Department of Pediatric Dentistry at the University Clinic of Dentistry in Vienna. Only children from 0 to 6 who presented with dental pain were included in the study group. After obtaining the caregivers’ consent for study participation, they were asked to answer the German version of the Early Childhood Oral Health Impact Scale (ECOHIS-G) questionnaire before treatment. 

The ECOHIS-G questionnaire consists of 13 questions and is divided into two main entities, the child impact section (CIS) (9 items) and the family impact section (FIS) (4 items). The child impact section comprises four subscales: symptoms (one item), child-related function (four items), psychology (two items), and self-image/social interaction (two items). The family impact section contains two subscales: parental distress (two items) and family-related function (two items). The questions inform about the frequency of certain events in the child’s life. Responses are given on an ordinal scale (0 = never, 1 = hardly ever, 2 = occasionally, 3 = often, 4 = very often). An additional answer option “do not know” is implemented in every item. If caregivers chose to respond with “do not know”, it was treated like a missing answer in the subsequent analysis [27]. Summing up the response codes for the questionnaire items generates domain scores and an ECOHIS sum score. If up to two responses on the child section or one answer on the family section were missing, an average of the remaining items for that section was imputed as a score for the missing items. The total score ranges from 0 to 52. A sum score of 0 indicates the absence of any problems, whereas a higher ECOHIS score indicated a greater impact of oral health problems on OHRQoL of preschool children and their families. The scores for the child and family sections have a possible range from 0 to 36 and from 0 to 16, respectively. In addition to the 13 items, the ECOHIS includes two questions asking the caregiver for a global rating of the child’s oral health and overall well-being. These global ratings have five response options (0 = excellent, 1 = very good, 2 = good, 3 = moderate, 4 = poor). Questionnaires were deemed invalid if more than five questions were not answered, if one or more answers within one subsection of the questionnaire were missing, if multiple answers were given to the same question, if more than two questions were answered by “don’t know” or if the patients’ literary or language skills were insufficient. 

After ECOHIS-G questionnaires were answered correspondingly and under the premise that the children’s’ compliance was sufficient, they were clinically examined for caries experience and plaque accumulation in accordance with WHO standards by calibrated pediatric dentists [29]. Caries status was assessed using the dmf-t index for deciduous teeth. In addition, the simplified additive plaque index by Ambjørnsen was used to assess plaque accumulation. A score of 1 represented visible plaque, whereas a score of 0 was documented when no plaque was visible. Moreover, the caregivers’ reason for presenting in the Emergency unit of the Department of Pediatric Dentistry and the children’s diagnosis was assessed. The documented causes for dental pain were not limited to ECC and its complications (irreversible pulpitis, apical periodontitis, swelling, fistulas), but in fact covered a variety of different options such as traumatic dental injuries, disturbances in tooth eruption, periodontitis, surgical concerns, or structural dental anomalies.

A control group comprised children from 0 to 6 years old free of dental pain who presented for routine control examinations. Control patients were chosen in correspondence with age and gender distribution of the study group. Hence, in every age group, the number of girls and boys was even in both collectives, allowing for good comparability. 

Statistical analysis was performed with IBM SPSS Statistics Version 27 (IBM Corp., Armonk, NY, USA). In advance, for a Pearson correlation coefficient of 0.30 and significance level of 0.05 (Power 0.90), a minimum sample size of 237 children was calculated. Descriptive statistics (mean, SD) were provided for continuous measurements (e.g., age). Nominal measurements (e.g., gender, type of diagnosis) are summarized using frequencies and proportions as well as crosstabulations. *t*-tests for independent samples were calculated to compare interesting proportions between both collectives. Variation analysis was performed to evaluate statistical significance and subsequently investigated for validity via correlation analysis. Correlation analysis was performed to analyze the influence of caries experience (dmf-t index), plaque accumulation, global oral health, and overall well-being on OHRQoL (ECOHIS scores). ECOHIS sum score was treated as a quasi-interval scaled. In dependance of the scaling of the compared parameters, different correlation coefficients were used (e.g., Pearson, Spearman, and Pointbiseral correlation). Additionally, Eta correlation, Chi-square test, and Phi-Cramer coefficient were calculated. Graphs were created using MS Excel^TM^ 2016 (IBM Corp., Armonk, NY, USA).

## 3. Results

Initially, the study group comprised 298 children with dental pain from 0 to 6 years old. However, 39 children (13.1%) were excluded due to incomplete records (dmf-t index, plaque index) or invalid questionnaires. Therefore, the results of only 259 children were considered for further analysis. A total of 141 patients were boys (54.4%) and 118 were girls (45.6%). The mean age was 4.2 years (SD ± 1.5). The results of the clinical screening showed a mean dmf-t score of 6.2 (SD ± 1.5) and mean dmf-s score of 12.5 (SD +/−13.5). Mean plaque accumulation was 64% (SD ± 0.5). The control group comprised 259 patients from 0 to 6 years old as well, of which 140 patients were boys (54.1%) and 119 were girls (45.9%). The mean age was 4.2 years (SD ± 1.5). The mean dmf-t score reached 3.9 (SD ± 4.8) and the mean dmfs-score 8.5 (SD ± 13). Mean plaque accumulation was 50% (SD ± 0.5). Table 1 summarizes the demographic and clinical data of both collectives. 

**Figure 1:**
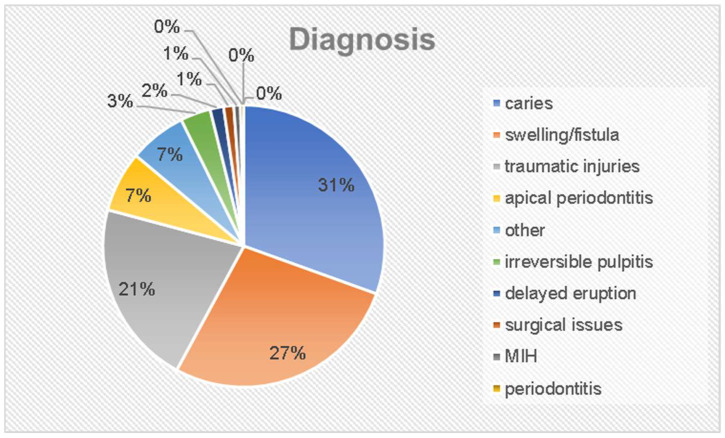
Diagnosis of children.

Most of the children of the study group presented without a medical referral (*n* = 230, 88.8%). A total of 5 patients (1.9%) were referred for ambulatory treatment, 3 (1.2%) for treatment under general anesthesia, and 21 (8.1%) without any further specifications. Most children of the study group presented with caries (*n* = 79, 30.5%) or complications of tooth decay such as irreversible pulpitis (*n* = 9, 3.5%), apical periodontitis (*n* = 18, 6.9%), swelling, or a fistula (*n* = 71; 27.4%). With 21.2% (*n* = 55), traumatic dental injuries were identified as the second most prevalent reason for medical consultation. Other children presented with abnormalities in their tooth eruption (*n* = 4; 1.5%), periodontitis (*n* = 1; 0.4%), and molar incisor hypermineralizations (*n* = 2; 0.8%). Only 1.2% (*n* = 3) sought surgical procedures. A total of 17 children (6.6%) had other complaints that had not been formally categorized on the study’s results sheet (Figure 1).

### 3.1. ECOHIS-G

**Figure 2:**
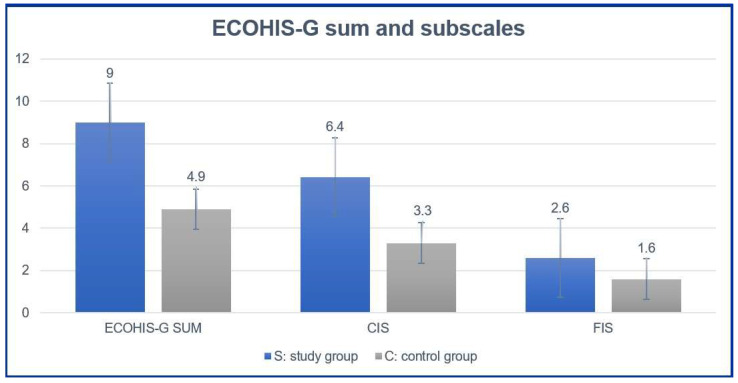
Comparison between ECOHIS-G sum and subscales between the study and control group.

With 9.0 (SD ± 7.4), the mean ECOHIS-G sum score in the study group was significantly higher compared to the control group, whose mean score only amounted to 4.9 (SD ± 5.6). Thus, children with dental pain showed a significantly lower oral health related quality of life than children without pain (*p* < 0.001). The lowest ECOHIS-G score in the study group was 0, the highest 40 (possible maximum = 52). In the control group, the lowest score equaled 0, whereas no patient showed a higher score than 27 (Figure 2). Apart from the item “missed preschool, day care or school” the study group scored higher in all other ECOHIS-G items. Statistically significant differences were observed in the items “had difficulty drinking hot or cold drinks”, “had difficulty eating some food”, “avoided smiling or laughing”, “been upset”, and “felt guilty”. The other items showed no significant differences between both groups. The lowest score in the study group was attributed to the items “avoided smiling or laughing” (0.2 ± 0.7) and “avoided talking” (0.2 ± 0.5). The highest score was observed when caregivers were asked about the frequency of pain in the children’s teeth, mouth, or jaws (2.0 ± 6.2) (Table 2). Naturally, the latter was identified to be the most compromised item. 

Analyzing the two subscales of the ECOHIS separately, the study group showed a mean CIS of 6.4 (SD ± 5.6), while the impact on children of the control group only scored 3.3 (SD ± 4.1). With a mean value of 2.6 (SD ± 2.8) FIS also reached a higher score in the study group (FIS_Control_: 1.6 ± 2.5). Hence, the difference between both groups was not only statistically significant in the ECOHIS-G sum score, but also in both subsections CIS and FIS (*p* < 0.05) (Figure 2 and Table 3).

In the study group, the global child’s oral health was rated as good (2.2 ± 1.2) and the overall well-being as very good (1.0 ± 1.0), whereas the control group rated its mean overall well-being as excellent (0.6 ± 0.1) and its global oral health as very good (1.2 ± 0.1). Equally in this regard, the difference between both groups was statistically significant (*p* < 0.001). The results imply that children suffering from dental pain are more likely to rate their global oral health and overall well-being worse than children without oral pain. 

### 3.2. Clinical Screening

A total of 213 (82.2%) children of the study group were diagnosed with caries and/or caries experience, while only 46 (17.8%) patients showed no cavities, fillings, or missing teeth. On average, dmf-t index amounted to six teeth. Four children showed generalized decay in 20 primary teeth. Due to little or no compliance carious, filled and/or missing surfaces could only be analyzed in 219 (84.6%) patients of the study group. On average, 12.5 (SD ± 13.5) teeth surfaces were carious, filled, and/or missing. In contrast, in 119 children (49.5%) of the control group, no caries, filled, or missing teeth were detected. On average, each child of the control group had four decayed, missing, or filled teeth (3.9 ± 4.8) and mean dmfs-score was 8.5 ± 13.0 (Table 4). Consequently, dmf-t index and dmf-s index showed significantly higher results in the study group (*p* < 0.001). With 63.7%, the mean plaque accumulation was also significantly higher in the study group compared to control patients (50.2%, *p* < 0.001). The results indicate that children with dental pain have more cavities and poorer oral hygiene than children free of such pain (Table 4).

### 3.3. Correlation Analysis

The relations between ECOHIS scores, global oral health, and overall well-being as well as dmf-t index and plaque accumulation were examined via correlation analysis. The overall well-being significantly correlated with CIS (r = 0.236, *p* = 0.000), FIS (r = 0.173, *p* = 0.005), and ECOHIS sum score (r = 0.382, *p* = 0.000). High rates of overall well-being indicate that the child’s quality of life is less likely to be compromised. The ratings of the child’s global oral health equally showed significant correlations with CIS (r = 0.478, *p* = 0.000), with FIS (r = 0.387, *p* = 0.000), and with ECOHIS sum score (r = 0.631, *p* = 0.000) (Table 5). These findings show that children whose oral health was rated higher also showed a higher quality of life. 

The dmf-t index and plaque accumulation significantly correlated with CIS and ECOHIS sum score. However, both parameters had no significant impact on FIS (Table 6). Moreover, children with caries experience and plaque showed significantly lower levels of global oral health (dmf-t: r = 0.573, *p* < 0.001, plaque index: *p* < 0.001) and overall well-being (dmf-t: r = 0.172, *p* = 0.006; plaque index: *p* = 0.028) (Table 6).

Further, the analysis of the correlation between different diagnoses and quality of life revealed that children with caries experience and caries-related complications such as irreversible pulpitis, apical periodontitis, swelling, and fistulas reached higher scores in the CIS (r = 0.381), the FIS (r = 0.326), and ECOHIS sum (r = 0.517) than children with no caries experience. This emphasizes the special role of caries experience in the context of OHRQoL. Global oral health also showed a significant correlation to the diagnosis of caries experience (*p* < 0.001) (Table 6). In contrast, the overall well-being showed no significant correlations with the diagnosis. Lastly, a strong correlation between the dmf-t index and the child’s diagnosis was observed (r = 0.626). Children with caries experience and related complications showed higher dmf-t scores than children with no caries experience. Another significant correlation was observed between the child’s referral status and plaque accumulation (*p* < 0.001). Children with plaque were more likely to present without a referral than children without plaque (*p* = 0.007). The presence of plaque and caries experience also proved to be strongly correlated (r = 0.505, *p* < 0.001). Children with poor oral hygiene were more prone to caries experience than children with good oral hygiene.

## 4. Discussion

Children worldwide suffer from dental pain, which not only affects the quality of life of the child but also their caregivers and families. It has a negative impact on their physical state, mental and social well-being, and causes disruptive sleep patterns and affects the child’s ability to learn [30]. Caregivers are often emotionally drained by feelings of guilt [31]. In the literature, only few reports address the explicit correlation of dental pain as a general symptom of different underlying causes and OHRQOL in preschool children.

As caries experience and untreated carious lesions are a frequent occurrence in the primary dentition and a prevalent cause for dental pain, the age group of 0- to 6-year-olds is of particular interest. Compared to previous studies on OHRQoL and caries experience, the age span of the recruited patients in the presented study is one of the most extensive. Pesaressi et al. studied 3-year-old children in Peru [32], Rajab et al. 4- to 5-year-olds in Jordan [33], and Randrianarivony et al. 3- to 5-year-olds children in Madagascar [34]. Children between 2 and 5 years old were examined in Chile [35] and Malaysia [36]. Antunes et al. covered 2- to 6-year-old children in Brazil [37]. The sample size of these studies varied from 130 to 1557 children [33,36]. The average sample size of similar studies examining the OHRQoL of children under the age of 6 was approximately 220 children. Thus, with 259 children, the sample size of the present study is above average.

The presented study found an association between dental pain and OHRQoL in preschool children, corroborating the findings of previous studies [2,3,38,39]. Children with tooth ache showed higher impacts in total ECOHIS score and in both subsections CIS and FIS, indicating that the child’s and their family’s quality of life were equally affected by the child’s oral health condition. With 9.0 (SD ± 7.4), the ECOHIS score of the study group was higher than in previous studies [2,38,39]. With the ECOHIS sum score of 4.9 (SD ± 5.6) in the control group, the difference between both groups was statistically significant (*p* < 0.001). Ortis et al. who recruited 534 children between 0 and 5 years old at the National Children’s Vaccination Day Program in Brazil reported an ECOHIS sum score of only 1.37 (SD ± 2.71). Nonetheless a statistically relevant correlation between dental pain and OHRQoL was established [2]. The study’s findings also agree with Clementino et al. on the correlation between dental pain and OHRQoL [39]. She examined 843 children from 3 to 5 years old in public or private preschools in Brazil. Fernandes measured tooth ache through the Dental Discomfort Questionnaire (DDQ-B) in 306 Brazilian children from 1 to 3 years old and correlated the results with ECOHIS scores (r = 0.441, *p* < 0.05), attesting that dental pain reduces OHRQoL [38]. All three cited studies from Brazil differed in methodic protocol to analyze the correlation of tooth ache and OHRQoL and in inclusion criteria for study participation. A significant difference lies in the fact that all three reports randomly recruited children who did not specifically suffer from dental pain. While the prevalence of tooth ache was 100% in the study group, the prevalence was only 10.1% [2] and 40.2% [38], respectively, in the mentioned publications. While Ortiz et al. and Clementino et al. asked one question to measure the presence of dental ache, Fernandes et al. used an entire questionnaire (DDQ-B).

Moreover, the analysis of ECOHIS subsections confirmed previous findings, which showed that the Child Impact Section (CIS) scored higher than the Family Impact Section (FIS) (CIS: 6.4 ± 5.6, FIS: 2.6 ± 2.8) [39]. In accordance with the results of Clementino et al., dental pain was identified as the most frequent problem in the CIS, while caregivers were most likely to feel guilty in the FIS [39]. The severity of dental pain and its impact on OHRQoL and overall well-being is underlined by the fact that eating food was identified as the second most prevalent problem for children. A well-balanced, nutritious diet is essential for children’s health development. This was followed by trouble sleeping in the presented study, whereas Clementino et al. identified difficulties drinking hot or cold drinks as the second most frequent problem for children. The second most frequent problem reported by caregivers was the financial burden of pediatric dental care, whereas Clementino reported that caregivers were mostly feeling upset [39]. In Austria, the costs of pediatric dental care are covered by the primary health care system. However, children suffering from dental pain are often diagnosed with ECC and require general anesthesia, which is not automatically covered by statutory health insurance.

Interestingly, despite high-quality dental care systems recent studies have shown that the prevalence of caries in preschool children lies at 35.1% in Austria [40] and 44.4% in Germany [41]. The global prevalence of caries in children is estimated between 35–45% in industrialized nations [40,41] and to be as high as 70% in developing countries [33,42]. With 82.2%, the share of children with caries experience was comparatively high in the study group. When compared to findings by Ortiz et al. who also examined the impact of dental pain on OHRQoL and used the dmfs-index for caries evaluation, a substantial discrepancy becomes evident. In their study, the mean caries prevalence was only estimated at 16.4%. The recruited children did not actually seek dental care on this particular day, but in fact were getting vaccinated. Given that therefore only 10.1% of the sample actually presented with dental pain and considering that dental pain was quantified by only one single question, comparability is limited [2]. Other studies that used ICDAS (International Caries Detection and Assessment System) to register caries reported a prevalence of 63.5% and 74.3% [38,39]. It is important to recognize that in contrast to the dmf-t index, the ICDAS also includes initial lesions and therefore leads to higher prevalence rates by nature [43]. Still, the presented caries prevalence was higher than in similar studies. An explanation lies in the fact that only children with dental pain were eligible for the participation in the study group. As dental caries presents as a primary reason for tooth ache, a higher prevalence can be expected in a patient collective that exclusively consists of children suffering from dental pain [8,44,45].

The analysis of the simplified plaque index by Ambjørnsen revealed that 63.7% of examined children showed plaque accumulation. Compared to previous findings in which 40.2% of children in Austria had poor oral hygiene, the presented share was evidently higher [27]. Once again, this significant difference derives from different inclusion criteria. While the presented study exclusively examined children with dental pain, Bekes et al. did not differentiate between children presenting with or without tooth ache [27]. Apart from the fact that the oral hygiene of children with tooth ache was worse than of children free of dental pain, the presented results also showed that children with poor oral hygiene were more prone to caries and/or caries experience than children with good oral hygiene. Having established that dental caries and its complications are the most prevalent causes for dental pain in children, the high share of plaque agrees with the linear cause–effect relationship of oral hygiene, caries, and dental pain. However, the linear rationale can turn into a vicious cycle rapidly increasing caries progression. Once children suffer from dental pain, oral hygiene often deteriorates further. Children’s compliance level decreases when brushing their teeth becomes associated with increasing pain.

A significant negative impact on CIS (r = 0.174, *p* = 0.005) and ECOHIS sum score (r = 0.183, *p* = 0.003) was observed. Other authors have confirmed that caries significantly reduces OHRQoL as well, as the observed ECOHIS sum scores increased [35,37,38]. In accordance with findings by Rajab et al., the presented results imply that caries experience has, however, no relevant influence on FIS [33]. Yet numerous previous studies reported a correlation between caries and all aspects of ECOHIS, including FIS [27,32,34,36]. Only Clementino et al. concluded that caries per se has no impact on OHRQoL [39]. The described ascertainments of the study reveal that there is still a need for the implementation of more adequate approaches to prevent, reduce, and control dental decay in preschool children. A promising approach in raising parental awareness lies in the introduction of obligatory dental consultations in the mother-and-child pass examinations during pregnancies and in the first months after childbirth.

By further analyzing the influence of dental pain on overall well-being and global oral health in the study group the multifaceted dimensions and wide-ranging implications of tooth ache become evident. In accordance with literature, the overall well-being was rated “very good” (1.0 ± 1.0), and global oral health was rated “good” (2.2 ± 1.2) [27,35]. The difference between the study group and the controls was significant (*p* < 0.001) in both categories. The assessment of overall well-being significantly impacted OHRQoL. If the overall well-being and global oral health were rated poorly, it was more likely that the child suffered from tooth ache and vice versa. This confirms previous findings that the overall well-being and oral health correlated with ECOHIS sum score and ECOHIS sub sections scores [27,34].

Consequently, the results imply that caries experience and poor oral hygiene significantly compromise the child’s OHRQoL, global oral health, and overall well-being. However, neither caries experience nor poor oral hygiene had a significant effect on the family’s quality of life.

## 5. Conclusions

The presented study offers a relevant perspective on a pressing public health issue by examining the interactions of dental pain and OHRQOL. The results may contribute to guiding dental health policies in defining and prioritizing a socially appropriate use of resources.

In accordance with international data, the study’s results highlight the importance of children’s oral health to their overall well-being and the profound impact oral health can have on children’s QoL. Children between the ages of 0 and 6 presenting with dental pain had a significantly worse OHRQoL than children without pain. Their compromised health proved to be significant in all ECOHIS items, namely both sub sections CIS and FIS and the overall ECOHIS score. Moreover, on average children with dental pain rated their global oral health and overall well-being significantly worse than children of the same age that regularly present for routine dental examinations. Caries experience and plaque accumulation correlated with the overall ECOHIS score and CIS.

For the future, a multicenter approach with the inclusion of rural areas is recommended in order to gain a more comprehensive analysis of dental pain and its impact on OHRQoL and to assess regional differences in Austria. The introduction of population-directed, obligatory dental consultations during pregnancies and in the first months after childbirth in the mother–child pass is a promising approach to increase parental awareness and reduce the prevalence of oral diseases in children at risk. The prevention and adequate management of dental pain requires knowledge on its predictors. Acknowledging that various disease patterns can lead to tooth ache, the vast majority of cases are, however, caused by tooth decay and its complications.

## Figures and Tables

**Table 1:** Descriptive analysis of demographic and clinical parameters of study and control group.

Clinical Data	Group	*n*	Mean Value	SD	Min	Max
age	study group	259	4.2	1.5	0	6
	control group	259	4.2	1.5	0	6
dmf-t	study group	259	6.2	5.1	0	20
	control group	259	3.9	4.8	0	20
dmf-s	study group	219	12.5	13.5	0	78
	control group	256	8.5	13.0	0	80
plaque-index	study group	259	64%	0.5	0	1
	control group	259	50%	0.5	0	1

**Table 2:** ECOHIS-G questionnaire mean value, standard deviations (SD), and *t*-test.

No.	Question	Mean Value S (SD)	Mean Value C (SD)	Difference S-C	*p*-Value
1	How often has you child had pain in his/her teeth, mouth or jaws?	2 (6.2)	1.6 (8.2)	0.4	0.457
How often has you child … due to dental problems or dental care?					
2	had difficulty drinking hot or cold drinks	0.8 (0.1)	0.3 (0.7)	0.5	<0.001
3	had difficulty eating some foods	1.1 (1.3)	0.4 (0.8)	0.7	<0.001
4	had difficulty pronouncing some words?	0.5 (1.0)	0.5 (1.0)	0	0.596
5	missed preschool, day care or school	0.9 (6.2)	1.5 (10.6)	−0.6	0 495
6	had trouble sleeping	1.1 (6.2)	0.4 (0.7)	0.7	0.061
7	been irritable or frustrated	1.1 (6.2)	0.8 (6.2)	0.3	0.604
8	avoided smiling or laughing	0.2 (0.7)	0.1 (0.4)	0.1	0.010
9	avoided talking	0.2 (0.5)	0.1 (0.4)	0.1	0.056
How often have you or another family member … because of your child’s dental problems or dental care?					
10	been upset	0.7 (1.1)	0.3 (0.8)	0.4	<0.001
11	felt guilty	0.9 (1.2)	0.5 (1.0)	0.4	0.001
12	taken time off of work	0.7 (1.0)	0.5 (0.9)	02	0.127
13	been financially impacted	0.8 (6.2)	0.2 (0.7)	0.6	0.166

S: study group, C: control group.

**Table 3:** ECOHIS-G subsections mean value, standard deviations (SD), and *t*-test.

ECOHIS Subsection	Mean Value S (SD)	Mean Value C (SD)	Difference S-C	*p*-Value
Child Impact Section (CIS)	6.4 (5.6)	3.3 (4.1)	3.1	<0.001
Family Impact Section (FIS)	2.6 (2.8)	1.6 (2.5)	1	<0.001
ECOHIS-G sum	9.0 (7.4)	4.9 (5.6)	4.1	<0.001

S: study group, C: control group.

**Table 4:** Comparison of clinical parameters between study (S) and control (C) group. Mean value, standard deviations (SD), and *t*-test.

Clinical Parameter	Mean Value (S) (SD)	Mean Value (C) (SD)	Difference S-C	*p*-Value
dmf-t	6.2 (5.1)	3.9 (4.8)	2.3	<0.001
dmf-s	12.5 (13.5)	8.5 (13.0)	4	<0.001
plaque	0.6 (0.5)	0.5 (0.5)	0.1	0.002

**Table 5:** Correlation between ECOHIS-G and overall well-being, global oral health, caries, and plaque.

Correlations		Child Impact Section (CIS)	Family Impact Section (FIS)	ECOHIS Sum
overall well-being	Spearman correlation	0.236	0.173	0.382
	*p*-value	0.000	0.005	0.000
global oral health	Spearman correlation	0.478	0.387	0.631
	*p*-value	0.000	0.000	0.000
dmf-t	Pearson correlation	0.174	−0.034	0.183
	*p*-value	0.005	0.584	0.003
plaque	Pearson correlation	0.173	−0.005	0.182
	*p*-value	0.005	0.940	0.003

**Table 6:** Correlation between dmf-t, plaque accumulation, and overall well-being and global oral health.

Correlation		Overall Well-Being	Global Oral Health
dmf-t	Spearman correlation	0.172	0.573
	*p*-value	0.006	<0.001
plaque	Chi2 correlation		
	*p*-value	0.028	<0.001

## Data Availability

All data supporting the reported results are available upon request from the corresponding author.
